# Life-course vaccinations for migrants and refugees: Drawing lessons from the COVID-19 vaccination campaigns

**DOI:** 10.7189/jogh.12.03064

**Published:** 2022-10-01

**Authors:** Silvia Declich, Giulia De Ponte, Giulia Marchetti, Maria Grazia Dente, Maria Elena Tosti, Lara Tavoschi, Pier Luigi Lopalco, Maria Laura Russo, Maurizio Marceca

**Affiliations:** 1 National Centre for Global Health, Istituto Superiore di Sanità, Rome, Italy; 2 Italian Society of Migration Medicine (SIMM), Italy; 3 Department of Translational Research and New Technologies in Medicine and Surgery, University of Pisa, Pisa, Italy; 4 Department of Biological and Environmental Sciences and Technologies, University of Salento, Lecce, Italy; 5 Department of Public Health and Infectious Diseases, Sapienza University of Rome, Rome, Italy

COVID-19 showed once more, and very evidently, that some disadvantaged subgroups, including migrants and refugees (M&Rs), are at higher risk of contracting a disease or suffering from its severe consequences in areas with high transmission [1,2]. This may be due to their living conditions, which make physical distancing difficult, and/or to their legal status, which may exclude them from health care services. Additionally, COVID-19 reminded us that M&Rs tend to also have suboptimal vaccination coverage compared to the general population due to several concurrent factors [3,4]:

- exclusion from health and vaccination plans and systems, often due to a lack of legal entitlements to health care or due to administrative/residence barriers;
- health system barriers due to language, lack of cultural sensitivity, lack of outreach and community engagement capacity, lack of collaboration with civil society organisations, barriers to primary care, and vaccination services access, including vaccination costs;
- high mobility of M&Rs;
- lack of confidence in the health system and misconceptions about the vaccine.

We propose some elements useful for orienting the research agenda and generating debate based on the experience of the COVID-19 pandemic. While M&Rs experienced exclusion due to the pandemic in many contexts, in others, it has been an opportunity not just to maximise coverage, but also to set up, test, and implement new, effective, and replicable approaches in vaccination services.

## ARGUMENTS FOR A FULL INCLUSION OF M&RS IN VACCINATION PLANS BEYOND COVID-19

Primary prevention interventions, including vaccination, are at the core of the public health response to promote health and prevent diseases. As shown above, socially disadvantaged groups, such as migrants, often benefit less from those interventions. Yet, multiple elements indicate that their immediate and full inclusion in vaccination plans during COVID-19 has been beneficial and should be transferred into routine vaccination plans.

In fact, including migrants in vaccination programmes not only protects this group, but also decreases the risk of further outbreaks in the community.

- Improving vaccination coverage at the national and global levels, like many preventive actions, is much cheaper and more effective in public health terms than efforts to control an epidemic (once transmission has been established) and to treat affected individuals, as demonstrated by COVID-19 [5].
- Vaccination should be considered as a health equity intervention: as some subgroups are at a disproportionately higher risk of contracting several diseases due to their social vulnerability, it becomes crucial that they are not excluded in the access to vaccines to avoid a second additional burden on their health, compared to more favoured groups.
- Universal access to available vaccinations throughout the life course is recommended for all (including M&Rs) by the Sustainable Development Goals (SDG) Target 3.8 “Achieve Universal Health Coverage, including financial risk protection, access to quality essential health-care services and access to safe, effective, quality and affordable essential medicines and vaccines for all”. In the case of COVID-19, equity in the access to vaccines was called for by the World Health Organization [6] and the European Centre for Disease Prevention and Control [7], among other institutions. Those calls can now be starting points for building inclusive vaccination plans beyond COVID-19.

## TOWARDS M&RS INCLUSION IN LIFE-COURSE VACCINATION SERVICES: A ROADMAP

The COVID-19 crisis’s offers an opportunity to transform prevention and vaccination approaches which should not be missed: based on the argument above, it is vital that the inclusive, free-of-charge, and proactive vaccination approaches tested during the pandemic are transferred to routine vaccination plans and services in the post-pandemic period.

Below we illustrate key building blocks for the inclusion of M&Rs in life-course vaccination services that can orient the direction of learning from and capitalization of experiences from COVID-19 in the post-pandemic context (**Photo**).

**Figure:**
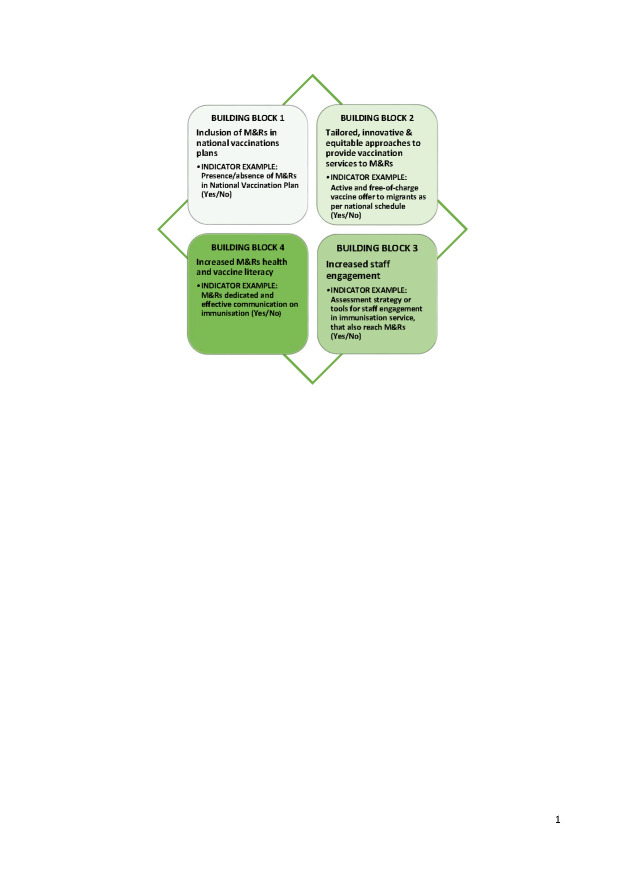
Photo: Building blocks for M&R inclusion in vaccination services and examples of monitoring indicators. Source: the authors’ personal collection.

### Building block 1: Inclusion of M&Rs in post-COVID-19 national vaccinations plans

- Inclusion of M&Rs in national vaccination plans beyond COVID-19, with the adoption (in terms of designing, financing, organising, and evaluating) of vaccination plans extended to the entire population, regardless of the legal status or other administrative barriers.
- Inclusion of M&Rs subgroups (e.g., M&Rs living in informal settlements or in camp-like settings, in first-line and second-line reception centres, in prisons and restricted communities, as well as the homeless and undocumented migrants) among the priority groups of vaccination plans related to diseases where social vulnerability implies a higher risk of infection, transmission, and negative outcome for the disease.

### Building block 2: Development of tailored, innovative, and equitable approaches for routine vaccination services targeting M&Rs

National and regional immunisation plans and guidelines should foresee strategies to overcome economic, organisational, and cultural barriers that hinder M&Rs’ access to and/or use of vaccination services, including mistrust, safety concerns, and vaccine hesitancy [4,8]. This may include:

- free-of-charge access to vaccination services at the point of delivery;
- providing a dedicated active offer of strategies responding to the groups’ specific risk factors, including adequate access to reservation and registration systems, as well as vaccination certificates as per national provision;
- strengthening health systems and primary healthcare providers, enhancing the accessibility, decentralisation, and outreach capacity of vaccination services to reach M&Rs (including newly arrived) living in communities, refugee camps, reception/detention centres, and prisons;
- fostering equity in health through innovative service delivery models: setting up, testing, and implementing mobile vaccination clinics, combined healthcare services (such as antenatal care and vaccination programmes), and mass vaccination [9];
- developing participatory approaches and engagement strategies to strengthen uptake, alongside innovative delivery mechanisms and tailored approaches to respond to specific determinants of M&Rs under-immunisation [10].

COVID-19 in many countries showed that trust of M&Rs in the health system is crucial. M&Rs may not come forward for vaccination in pandemic or outbreak situations, even if entitled to, if they have experienced exclusion or stigma in previous encounters with health care services. Generally, M&R’s trust in the system needs to be built since arrival through systemic interventions, such as:

- firewalls to shield migrants in irregular situations from the possible transfer of their personal data to immigration authorities when they attempt to access health-care services with outreach campaigns to inform migrants in irregular situations [11];
- access to primary care systems, especially registration with general practicioners, also beyond vaccination;
- use of trusted groups or sources (non-governmental organizations, community groups) for communication; increased funding for and collaboration with these trusted groups;
- avoidance of labelling specific groups as “infectious” or “hesitant” to avoid increasing stigmatisation and distrust [12].

### Building block 3: Increased engagement of staff and cultural competence of the system

- Increasing cultural sensitivity and competence for health personnel and the whole health system [1,13].
- Educating primary care staff on the M&R’s right to access healthcare [12].
- Supporting the health literacy of public health systems' immunization services; reorganizing and refocusing immunization services by making them easier and more equitable for M&Rs to navigate; understanding and using information and services to take care of their health and access immunization programs [14].
- Strengthening the communication capacities of vaccination services, including their engagement capacities with communities and their respected leaders, to ensure that messaging on vaccination is culturally and linguistically appropriate.
- Five interventions have been identified to improve cultural competence in healthcare systems: 1) developing programmes that recruit staff who reflect the cultural diversity of the community served; 2) using interpreter services or bilingual providers for patients with limited native language proficiency; 3) training healthcare providers in cultural competence; 4) using linguistically and culturally appropriate health education materials; and 5) creating culturally specific healthcare settings [15].

### Building block 4: Increased M&Rs’ vaccine literacy

Establishing vaccine literacy education programmes and strategies to promote vaccine confidence and vaccine uptake among M&Rs [10,16].

- Offering health promotion educational interventions through a community-based approach.
- Vaccine literacy should consider both the individual’s level of health literacy and the complexities of the contexts within which people act [17]. However, comprehensive, adequate, accessible, and language & culturally friendly information and education materials on preventive measures tailored to migrants are not common [18].

## MONITORING PROGRESS OF INCLUSIVE VACCINATIONS, BASED ON A HEALTH EQUITY PERSPECTIVE

Progress in the inclusion of socially vulnerable groups in vaccination, with specific reference to M&Rs, needs to be monitored at national and regional levels through:

- setting up strategic goals, targets, and indicators for national vaccination plans to allow for monitoring of progress and impact based on health equity and public health perspective (**Photo**);
- setting up or expanding immunisation information systems with appropriate disaggregation by key social determinants of health, according to a set of core variables in relation to the migration status [19];
- setting an appropriate vaccination coverage target related to M&Rs, at least for diseases targeted for elimination or eradication (e.g., polio and measles) to be used as a proxy for inclusiveness.

## CONCLUSIONS

COVID-19 vaccination campaigns across countries are unmissable opportunities to learn and capitalise on experiences in designing, testing, and implementing new approaches in primary prevention and vaccination services that are effective and replicable for routine vaccinations, including catch-up efforts. A specific focus on the inclusion of M&Rs is key to ensuring the availability and equity of vaccination services. We consider that the building blocks presented above can be useful to orient both the post-pandemic research agenda on the subject and policy development of national health systems, as well as to stimulate further initiatives and collaborations.
